# Development and Implementation of Proton Therapy for Hodgkin Lymphoma: Challenges and Perspectives

**DOI:** 10.3390/cancers13153744

**Published:** 2021-07-26

**Authors:** Pierre Loap, Ludovic De Marzi, Alfredo Mirandola, Remi Dendale, Alberto Iannalfi, Viviana Vitolo, Amelia Barcellini, Andrea Riccardo Filippi, Barbara Alicja Jereczek-Fossa, Youlia Kirova, Ester Orlandi

**Affiliations:** 1 Department of Radiation Oncology, Institut Curie, 75005 Paris, France; ludovic.demarzi@curie.fr (L.D.M.); remi.dendale@curie.fr (R.D.); Youlia.kirova@curie.fr (Y.K.); 2 Radiation Oncology Clinical Department, National Center for Oncological Hadrontherapy (CNAO), 27100 Pavia, Italy; alfredo.mirandola@cnao.it (A.M.); alberto.iannalfi@cnao.it (A.I.); viviana.vitolo@cnao.it (V.V.); amelia.barcellini@cnao.it (A.B.); ester.orlandi@cnao.it (E.O.); 3 Institut Curie, PSL Research University, University Paris Saclay, INSERM LITO, 91400 Orsay, France; 4 Radiation Oncology Department, Fondazione IRCCS Policlinico San Matteo and University of Pavia, 27100 Pavia, Italy; a.filippi@smatteo.pv.it; 5 Department of Oncology and Hemato-Oncology, University of Milan, 20122 Milan, Italy; barbara.jereczek@ieo.it; 6 Division of Radiotherapy, IEO European Institute of Oncology IRCCS, 20141 Milan, Italy

**Keywords:** Hodgkin lymphoma, proton therapy, NTCP model, toxicity

## Abstract

**Simple Summary:**

Hodgkin lymphoma (HL) is a highly curable disease; proton therapy for mediastinal HL irradiation might theoretically reduce late toxicities compared with classical radiotherapy techniques. However, optimal patient selection for this technique is subject to debate. While implementation at a larger scale of proton therapy for HL may face organizational, political, and societal challenges, new highly effective systematic drugs are being widely evaluated for this disease.

**Abstract:**

Consolidative radiation therapy for early-stage Hodgkin lymphoma (HL) improves progression-free survival. Unfortunately, first-generation techniques, relying on large irradiation fields, were associated with an increased risk of secondary cancers, and of cardiac and lung toxicity. Fortunately, the use of smaller target volumes combined with technological advances in treatment techniques currently allows efficient organs-at-risk sparing without altering tumoral control. Recently, proton therapy has been evaluated for mediastinal HL treatment due to its potential to significantly reduce the dose to organs-at-risk, such as cardiac substructures. This is expected to limit late radiation-induced toxicity and possibly, second-neoplasm risk, compared with last-generation intensity-modulated radiation therapy. However, the democratization of this new technique faces multiple issues. Determination of which patient may benefit the most from proton therapy is subject to intense debate. The development of new effective systemic chemotherapy and organizational, societal, and political considerations might represent impediments to the larger-scale implementation of HL proton therapy. Based on the current literature, this critical review aims to discuss current challenges and controversies that may impede the larger-scale implementation of mediastinal HL proton therapy.

## 1. Introduction

Hodgkin lymphoma (HL) is characterized by a high curative rate, evaluated between 80% and 90% [1]. Consolidative radiation therapy is currently the gold standard for early-stage HL since it is associated with improved progression-free survival [2]. However, first-generation radiation therapy techniques, using large extended fields, increased cardiac and pulmonary morbidity as well as secondary cancers for mediastinal HL irradiation [3]. Fortunately, technical progress was subsequently made to better spare organs-at-risks (OAR) without altering the local tumor control or patient, such as the development of intensity-modulated radiation therapy (IMRT) with smaller target volumes [4] or respiratory control techniques [5]. Due to the physical characteristic of proton beams to deliver most of their energy at the end of their trajectory, proton therapy has been evaluated for mediastinal HL irradiation to spare OARs [6]. It has been demonstrated that proton therapy could spare OAR significantly better than other static or rotational IMRT techniques [7], which may translate into fewer late cardiac, pulmonary, esophageal, and thyroid late toxicities. In some cases, this reduction may also lead to a reduction in the secondary cancer risk [8], even if, according to current models, the risk related to fractionated low doses has been recently reevaluated [9,10]. Proton therapy is consequently a promising technique for selected patients who may benefit from maximal OAR sparing, such as young female patients or patients with cardiovascular comorbidities. However, while proton therapy for HL is promising to limit late toxicity, its implementation in clinical practice is facing multiple issues. Prioritization methods for patient selection are subject to debate, considering the current particle beam therapy facility shortage and the societal and operational cost of proton therapy compared with lower-cost classic radiotherapy [11]. Further, management of HL has recently been rapidly changing with the evaluation of new, highly therapeutic systemic drugs (such as brentuximab vedotin and anti-CD30 car-T cells [12,13]). The purpose of this review is to identify and discuss controversies and challenges for the larger implementation of this promising technique in the near future.

## 2. Patient Selection for Proton Therapy: Current Approaches and Limitations

**Figure 1:**
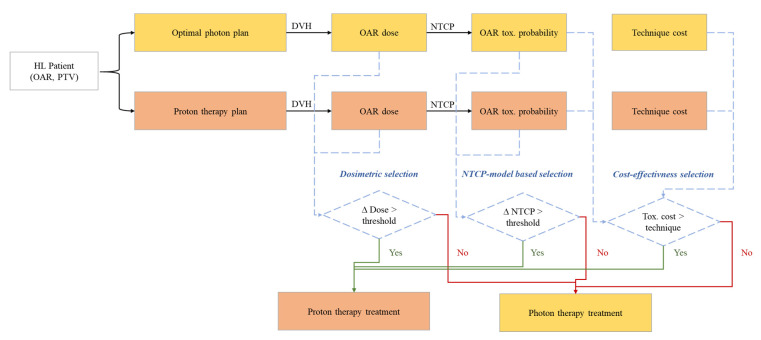
Current approaches to select patients for proton therapy. All selection processes rely on a dosimetric comparison between optimal photon (usually VMAT) and proton therapy plans. Depending on the methods, patients could be directly selected based on the dosimetric comparison (“dosimetric selection”), after evaluation of predicted toxicity reduction with proton therapy based on NTCP models (“NTCP model-based selection”); or after cost-effectiveness calculations (“cost-effectiveness selection”). HL: Hodgkin lymphoma; OAR: organ-at-risk; PTV: planned target volume; DVH: dose-volume histogram; NTCP: normal tissue complication probability; ∆: difference.

Multiple strategies have been proposed to select patients for proton therapy. These approaches can be based on dosimetric parameters, normal tissue complication probability (NTCP) models, or cost-effectiveness considerations (Figure 1).

### 2.1. A Dosimetric-Based Approach

#### 2.1.1. Theoretical Principle

In the context of limited resources, a selection process should logically take place to reserve proton therapy for the HL patients who would benefit the most from this technique. The ILROG guideline consensus about proton therapy for HL patients [14] currently recommends that, before treating an HL patient with proton beams, the radiation oncologist must demonstrate that it provides a benefit to the patient compared with an optimally planned photon therapy technique. According to these recommendations, the optimal proton therapy technique should be an involved site (ISRT) or involved node (INRT) radiation therapy, using pencil beam scanning, and considering the respiratory movement with DIBH or 4D CT scans. The treatment plan should be optimized to avoid MHD > 5 Gy, MLD > 10 Gy, or mean dose to the breast > 4 Gy. 

Current ILROG guidelines for HL radiotherapy [4] recommend planning with DIBH-IMRT, using ISRT or INRT if available, and image-guided radiotherapy (IGRT). Fiandra et al. [15] compared the dosimetric properties of diverse IMRT techniques including helical tomotherapy (HT), single arc VMAT, and “butterfly” multiple arc VMAT (B-VMAT); they concluded that HT and B-VMAT would achieve the optimal compromise between target conformation and OAR sparing. Consequently, while dosimetric comparison between proton therapy and optimal photon technique should rely on ISRT (or INRT) planning with a respiratory control, the optimal photon technique (“butterfly” IMRT, VMAT, B-VMAT, or HT) for comparison is still subject to debate. In clinical practice, an optimal technique cannot generally be pre-defined, each patient being unique in terms of anatomical disease presentation, age, risk factors, and chemotherapy regimen.

#### 2.1.2. Application in Clinical Practice

Based on the dosimetric comparison between proton therapy and optimally planned IMRT, dose to OAR and planned target volumes coverture can be compared. A patient selection can be made at this level based on the absolute dosimetric gain.

Based on a retrospective analysis of HL patients treated with proton therapy, dosimetric selection criteria could be proposed [16]. Such criteria could be adapted to the patient clinical presentation (considering age, sex, or comorbidities) and history (such as chemotherapy regimens, BRCA mutations). For informative purposes, dosimetric selection criteria for HL proton therapy at the Institut Curie (Paris, France) are provided in Table 1.

Based on a cohort of 21 patients treated with DIBH IMPT, Ntentas et al. [17] noted the dose reduction to the heart, the breast, the lung, the spinal cord, and the esophagus, but increased hotspots compared with VMAT. They observed that the dosimetric advantage of proton therapy in terms of MHD and mean dose to the breast was observed overall when clinical treatment volumes extended below the 7th thoracic level and for female patients with axillary disease. Definition of such patient groups, who are expected to benefit the most from proton therapy, might lead to direct proton therapy selection without dosimetric comparison with IMRT in the future.

#### 2.1.3. Advantages and Limitations

This selection method is simple to implement but presents some limitations. A dosimetric gain is not necessarily informative on the potential clinical benefit for the patient, as evidenced by non-significant or low toxicity gain when applying NTCP models as discussed in the first section [18,19]. The uncertainties with RBE modeling are a matter of concern. Based on NTCP models, Marteinsdottir et al. [20] found that toxicity estimation might be underestimated in dosimetric studies when using a fixed RBE value for proton beams and differences might become non-significant when a variable RBE value is considered.

### 2.2. A NTCP-Model-Based Approach

#### 2.2.1. Selection Based on Expected Toxicity Reduction

Dosimetric superiority does not necessarily translate into clinical benefit; consequently, NTCP models have been used to predict expected radiation-induced toxicity reduction to ultimately select patients who may benefit the most from proton therapy. Langendjik et al. [21] proposed a stepwise methodology for patient selection for protons based on an in-silico comparison between toxicity predicted by NTCP models with proton therapy and optimal photon radiotherapy; such comparative approach has recently been accepted by the Dutch health authorities to allow proton therapy reimbursement. It should be stressed that this NTCP model-based method only applies when the primary goal of proton therapy is to reduce toxicity, which is effectively the case for HL. Optimal VMAT and IMPT plans are simultaneously generated for each evaluated patient. The absolute dosimetric differences to each OAR are retrieved and converted into a toxicity reduction probability based on various NTCP models adapted to the corresponding OAR (∆NTCP). When this toxicity reduction probability is greater than a predetermined threshold (which is usually subjective), the patient is accepted for proton therapy treatment. This selection method has recently demonstrated its feasibility for head and neck cancer patients in the Netherlands [22].

Scorsetti et al. [8] proposed a similar approach specifically for mediastinal HL patients; the authors considered as relevant late adverse events radiation pneumonitis, esophagitis, and cardiac mortality (toxicity probability, estimated with NTCP models), secondary cancer (excess absolute risk (EAR), estimated with Schneider model), and ischemic heart disease and left ventricle failure (relative risk increase, estimated with regression models). Multiple arbitrary thresholds were proposed: an NTCP composite score for VMAT (equal to the sum of NTCP model-based probabilities for cardiac mortality, pneumonitis, and esophagitis) > 8% (higher threshold) or >5% (lower threshold); a secondary cancer EAR composite score with VMAT (equal to the sum of all cancer EAR) > 15 (higher threshold) or >10 (lower threshold) per 10,000 patients-year, and a cardiotoxicity RR composite score with VMAT (equal to the sum of RR for IHD and LV failure) > 0.25 (higher threshold) or >0.10 (lower threshold). Based on these NTCP, EAR, and RR composite score thresholds, the author evaluated the proportion of eligible patients to IMPT based on diverse selection rules, applied on a cohort of 20 patients: (1) if one considered that HL patients are eligible for proton therapy when the three composite scores with VMAT planning were simultaneously greater than the higher thresholds: 5% patients were eligible for IMPT; (2) if HL patients were eligible for proton therapy when the three composite scores with VMAT planning were simultaneously greater than lower thresholds, 20% patients were eligible for IMPT; (3) if HL patients were eligible for proton therapy when composite scores excessed one higher threshold or two lower thresholds, 75% of the patients would eligible for IMPT. It should be underlined those toxicities were not weighted in the calculation of NTCP, EAR, or RR composite scores and that the proposed thresholds did not concern toxicity difference between VMAT and IMPT (but toxicity prediction with VMAT only).

When a patient can develop multiple late toxicities and when the clinical decision is based on a composite score, the question of differentially weighting toxicities should be considered: all OAR late toxicities do not have the same functional impact or prognosis for the patient. Carbini et al. [23] proposed a weighted toxicity score (WTS) for tyrosine-kinase inhibitors (TKI) evaluating multiple weighting strategies based on CTCAE toxicity grade (linear ponderation, binary, weak exponential, strong exponential, and clinician defined). TKI dose reduction is best correlated with the clinical-weighted model. Such an approach could probably be developed for HL lymphoma radiotherapy, for which a WTS could be calculated on competitive treatment plans between VMAT and IMPT, from which an indication for IMPT could be taken.

#### 2.2.2. Limitations of NTCP Models

While the NTCP model may probably be more clinically relevant than crude dosimetric parameters, some pitfalls associated with NTCP calculation must be kept in mind.

First and foremost, NTCP models have been retrospectively developed with a given radiotherapy technique, at a precise moment. Troeller et al. [24] unambiguously demonstrated that 3D-RT-based NTCP models imperfectly work for IMRT. The validity of applying NTCP models that have been based on photon techniques for IMPT irradiation is uncertain; DNA damage with proton therapy is not fully understood but is different from classical photon beam radiobiological effects and might differ between passive scattering and pencil beam scanning proton beams [25]. Mee et al. [26] stressed that NTCP models should theoretically be adjusted with the acquisition of new clinical data and updating of existing databases (which poses the problem of observation delay); finally, ∆NTCP thresholds could be prospectively adjusted based on clinical feedback and on increasing proton therapy treatment capacity. Consequently, patients that might not have been accepted for proton therapy at a given moment could have been selected later, and inversely. However, trials could be proposed for patients that fail to meet the ∆NTCP threshold. On the other hand, it could be mentioned that any attempt to implement an NTCP model-based approach failed for lung cancer due to too small NTCP differences between protons and photons [27]. Oinam et al. [28] demonstrated that different NTCP calculation models, such as LKB or Niemerko model, could lead to significantly different toxicity predictions. Finally, most NTCP models disregard patient clinical characteristics; however, Köthe et al. [29] demonstrated for ocular toxicity of proton therapy that considering additional clinical variables such as age, tumor involvement, HTA, or sex, could substantially increase the performance of the NTCP models. This is particularly important for HL proton therapy since the expected benefit of proton therapy depends on the clinical features of the patient: to exemplify, patients with multiple cardiovascular risk factors are expected to benefit the most from heart sparing.

In addition, the proposition of the selection threshold is subjective and consequently debatable. Using three different selection rules based on unweighted toxicity scores, Scorsetti et al. [8] demonstrated that the proportion of patients eligible for HL proton therapy could vary from 5% to 70%. While Vaishampayan [30] underlined that while WTS had the advantage of being easy to comprehend, ponderation could be arduous between acute and long-term toxicity. The WTS concept was developed for TKI, and adaptation should have to be made for HL radiotherapy. Finally, toxicity weighting is usually done by the treating physicians; however, Cheung et al. [31] demonstrated that there were major discordances between physician perception and patient experiences and expectations. For HL, an a priori evaluation of patient expectations concerning radiotherapy-related toxicities could help weighting toxicity when developing a potential patient-tailored composite score.

#### 2.2.3. Towards a Life-Year-Lost Approach

Further approaches have been proposed to weigh late radiation-induced toxicities by taking into account the related life-year-lost (LYL) estimate. Rechner et al. [19] compared the LYL between DIBH and FB IMPT and IMRT, based on regression models from dose-volume distribution to different OAR. Considered adverse events were heart failure, myocardial infarction, valvular heart disease, lung cancer, and breast cancer. They found that LYL related to late treatment-related toxicity was 2.1 years for FB-IMRT, 1.3 years for DIBH-IMRT, 0.9 years for FB-IMPT, and 0.9 years for DIBH-IMPT. Of note, the LYL was comparable between DIBH-IMRT and FB-IMPT or DIBH-IMPT. LYL was mainly driven by lung cancer and valvular disease. Consequently, since the predicted treatment-related mortality is low for HL with modern radiation techniques and that LYL disregards non-fatal functional morbidity, a potential proposition could be to compare quality-adjusted life-year lost (QALYL) estimates between IMPT and IMRT. Such an approach has already been evaluated by Brodin et al. [32] for head and neck cancer.

### 2.3. A Cost-Effectiveness Approach

#### 2.3.1. Current Evaluations

To take account of the financial cost of HL proton therapy compared with standard radiotherapy, some cost-effectiveness models are currently being developed for HL patients. Vega [33,34] evaluated on a cohort of 40 HL patients the cost-effectiveness of proton therapy for coronary heart disease reduction. This study was based on the Framingham cohort for baseline risk and used a Markov chain model. It appeared that HL proton therapy was cost-effective for 50% of women and 60% of men if the willingness to pay (WTP) was 100,000$ per quality-adjusted life-year (QALY), and for 60% of women vs. 73% of men if the WTP was 200,000$ per QALY. With a WTP of 100,000$ per QALY, an MHD reduction of 5 Gy, 4 Gy, and 3 Gy yielded acceptability in 100%, 75%, and 38%, respectively; with a WTP of 200,000$ per QALY, an MHD reduction of 5 Gy, 4 Gy, 3 Gy, and 2 Gy yielded acceptability in 100%, 100%, 100%, and 75% respectively. However, this study did not consider secondary cancers, pulmonary toxicity, or other cardiac toxicity such as congestive heart failure or VHD; it is consequently expected that HL proton therapy would be more cost-effective than the estimations of this study. It should finally be noted that there are ongoing efforts by the PTCOG lymphoma subcommittee to conduct a large-scale cost-effectiveness study for HL proton therapy.

#### 2.3.2. How to Democratize HL Proton Therapy?

To increase the cost-effectiveness of HL proton therapy, systemic improvements must be made. Bortfeld et al. [35] considered that democratization of hadrontherapy required a faster delivery system, better integration in cancer centers, effective workflow, use of hypofractionated regimens when possible, and scaling down proton therapy system size. The possibility to use a specific treatment chair to accelerate the delivery procedure and to avoid the complexity of a gantry system has been extensively studied and discussed. Sheng et al. [36] evaluated the performances of a specific treatment chair with six degrees of freedom that may suppress the need for a gantry. Such a device could be used for HL proton therapy. Finally, FLASH proton therapy [37] might provide a way to accelerate proton therapy treatment, but this is yet to be evaluated on patients.

#### 2.3.3. Making HL Proton Therapy Financially Sustainable: A Challenge

The financial sustainability of particle radiotherapy is challenged by the commissioning of effective photon therapy techniques which might lead to high WTP per QALY to be cost-effective for proton therapy. A recent dosimetric study by Moreno et al. [38] evaluated 57 OAR dosimetric parameters between IMRT and proton beam radiotherapy: DIBH-IMRT yielded comparable results with 65% of parameters with FB-double scattering (DS) proton therapy, 56% of FB-IMPT, and 53% of DIBH-DS. Notably, IMRT was superior to proton therapy for high dose sparing. The optimization of the IMRT technique consequently represents a challenge for the financial sustainability of proton therapy in cost-effectiveness analyses.

To ease HL proton therapy treatment the necessity of DIBH has been questioned by some authors. Everett et al. [39] demonstrated that DIBH for IMPT did not reduce MHD but only MLD; they confirmed the superiority of IMPT over DIBH-IMRT. DIBH might consequently not be necessary for HL proton therapy but to consider the sensibility of proton beams to movement, FB would have to be associated with robust optimization and 4D CT [40,41] possibly combined with rescanning technique [42], which is equally complex to implement in clinical practice.

## 3. Future Implementation of HL Proton Therapy: Challenges and Pitfalls

**Figure 2:**
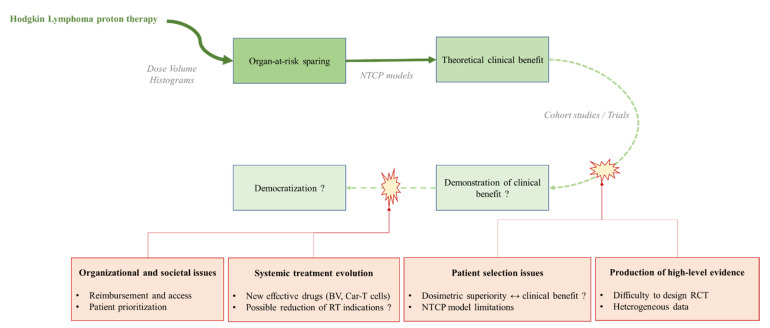
Current challenges for wider Hodgkin lymphoma (HL) implementation. The theoretical benefit of proton therapy for Hodgkin lymphoma in terms of toxicity reduction is accepted (based on dosimetric comparisons or NTCP models); however, production of high-level evidence is complex: HL is a rare disease for which late radiation-induced toxicity evaluation requires long-term follow-up. In addition, the optimal patient selection process is subject to debate, since dosimetric superiority does not necessarily translate into clinical benefit and that NTCP models have limitations. Furthermore, new highly effective treatments are being evaluated, such as Brentuximab Vedotin (VD, for frontline treatment) or anti-CD30 car-T cells (for relapse/refractory disease) which might possibly lead to a reduction of RT indications in HL management in the future. Organizational issues (such as prioritization of patients in a proton therapy center) and societal issues (reimbursement considerations) have also to be considered to implement HL proton therapy at a larger scale.

Figure 2 provides a synthetic view of the current challenges for implementing Hodgkin lymphoma proton therapy at a larger scale.

### 3.1. Changes in Hodgkin Lymphoma Treatment Paradigm

#### 3.1.1. Limitation of Radiation Therapy Indications in HL: A General Trend

There is currently a global worldwide trend to reduce the dose and the consolidative radiotherapy indications in HL management, which might be motivated by fear of late adverse events and the development of new effective drugs that might challenge radiotherapy. The HD10 trial [43] demonstrated the clinical equivalence of 20 Gy compared with 30 Gy consolidative radiotherapy after four ABVD chemotherapy cycles for favorable low-risk HL. Later, the RAPID Trial [44] and the H10 trial [2] tried to suppress radiotherapy after an interim PET negative for localized HL but failed to demonstrate non-inferiority of radiotherapy sparing in such a situation. However, the recent GHSG HD17 study was in favor of chemotherapy alone for early-stage unfavorable patients with a negative PET reevaluation after four chemotherapy cycles [45]. Omission of RT for advanced HL patients with PET-negative evaluation after ABVD chemotherapy has been evaluated in the FITIL/FIL HD 0607 trial [46]. In this study, patients with stage IIX, III, or IV HL were treated with two cycles of ABVD; those who were interim PET-negative received four additional cycles of ABVD while those who were interim PET-positive received escalated BEACOPP. Patients with interim and end-of-treatment PET-negative evaluation were randomized to consolidation RT or no further treatment. No differences in PFS or OS were observed at a 5-year follow-up. The phase-2 RAFTING trial is currently evaluating a risk-adapted and response-adapted approach for radiation therapy omission based on total metabolic volume and early PET response; patients at higher risk of relapse would receive radiation therapy in combination with immunotherapy (NCT04866654). PET negativity may thus identify patient populations of newly diagnosed HL patients who may not require consolidation RT. An alternative approach to limit radiation exposure currently under investigation is the reduction of irradiation field sizes with photon RT techniques. Currently, ISRT and INRT usually consider as target volumes all the initially involved sites, whether or not these sites have responded to chemotherapy. Limiting radiation fields to the residual sites only (residual site radiation therapy, RSRT) has been evaluated for advanced-stage HL in the HD15 study [47] and, more recently, for early-stage HL, by investigators from the Memorial Sloan Kettering Cancer Center: Kumar et al. evaluated an RSRT strategy for bulky HL patients (IIA/BX) following 4 cycles of brentuximab vedotin, doxorubicin, vinblastine, and dacarbazine (BV-AVD) [12]. After initial chemotherapy, patients who were considered negative, according to PET imaging or biopsy, were randomized assigned to 4 different treatment groups: 30 Gy ISRT (cohort 1), 20 Gy ISRT (cohort 2), 30 Gy RSRT (cohort 3), or no further treatment (cohort 4). Two-year progression-free survivals were 93%, 97%, 90%, and 97%, for cohorts 1 to 4, respectively, suggesting that achievement of a complete response after initial chemotherapy with BV-AVD regimen could be associated with an excellent short-term outcome with RSRT or without RT.

#### 3.1.2. Development of New Effective Systemic Treatments

Brentuximab Vedotin (BV) has been recently evaluated in the frontline context. Kumar et al. [48] found that four cycles of BV-AVD and 30 Gy involved-site radiotherapy yielded a 93.3% 1 year- for unfavorable early-stage HL. A subsequent multicenter study on 117 patients [12] similarly evaluating four BV-AVD cycles for unfavorable early-stage HL including bulky disease demonstrated comparable 2-year PFS with or without consolidative involved-site radiation therapy (93% and 97% respectively) and consequently proposed elimination of consolidative irradiation after complete metabolic response with this chemotherapy regimen. Metzger et al. [49] demonstrated that replacing vincristine with BV in frontline pediatric OEPA or COPDac protocols could spare consolidative radiotherapy for 35% of the children. In addition, multiple immunotherapies are currently being evaluated in the context of r/r HL, with encouraging results. Nivolumab, Pembrolizumab, Sintilimab, Tislelizumab, which are anti-PD1 antibodies, have been respectively evaluated in the Checkmate 205 [50], KEYNOTE-087 [51], ORIENT-1 [52] and BGB-A317-203 [53] trials with overall response rates (ORR) of 69%, 72%, 80% and 86%, respectively. Other notable immunotherapies currently under investigation for r/r HL include Camidanlumab Tesirine, an anti-CD25 antibody–drug conjugate, associated with an ORR of 71% [54], and AFM13, a CD16A/CD30 bispecific antibody, which yielded an ORR of 83% in combination with Pembrolizumab [55]. Finally, Ramos et al. [13] reported on a cohort of 41 patients a promising 94% 1-year OS following administration of anti-CD30 car-T cells after fludarabine-based lymphodepletion.

### 3.2. Organizational and Societal Challenges

#### 3.2.1. Reimbursement Issues

HL proton therapy faces worldwide reimbursement issues, which heavily depend on the country’s insurance system. To exemplify this diversity, the Dutch health system currently reimburses HL proton therapy when an expected clinical benefit is demonstrated based on NTCP models [21]. The French health system systematically reimburses HL proton therapy for children and teenagers (until 18 years old) [56] but not for adult patients, despite common long-term toxicity issues between children and young adults. In the United States, the reimbursement depends on the patient’s insurance coverage. Kerstiens et al. [57] concluded that the expansion of proton therapy centers was not sustainable under the current reimbursement model and that coverage decisions made by insurers would lead to the reduction of economically viable proton therapy centers. In this context, international cooperation could be considered to ensure that HL patients might benefit from proton therapy when their clinical situation justifies it.

#### 3.2.2. Access to Proton Therapy Centers

The inequality of access at a national and international level represents an additional challenge for larger-scale HL proton therapy implementation. Belard et al. [58] tested the feasibility of telemedicine for proton therapy. The European Particle Therapy Network, created in 2015 for clinical and translational research at a European level between the 20 proton therapy facilities [59], aims to increase the current evidence level of particle therapy clinical research [60]; this will undoubtedly prove useful for rare diseases such as HL.

#### 3.2.3. Prioritization patients in a proton therapy center

Finally, at the proton therapy center level, the capacity of treatment might be limited by the number of the treatment room. With limited disponibility, HL might not be prioritized over other indications for proton therapy for which the level of evidence of particle therapy is higher (such as pediatric tumors, uveal melanomas, skull-base tumors) and over patients with in-place tumors [16,61].

## 4. Conclusions

The development of proton therapy for HL is currently facing multiple challenges. Optimal patient selection for HL proton therapy is complex and subject to intense debate. In addition, one should acknowledge the accelerating development of new effective systemic treatments for HL disease. Furthermore, technical, organizational, and societal questions will have to be tackled to implement at a larger scale proton therapy for mediastinal HL.

Nevertheless, proton therapy should theoretically reduce secondary malignancies and late radiation-induced toxicities thanks to its minimal distant-to-target dose deposition. While selection criteria are yet to be precisely defined, specific patient populations may readily benefit from proton therapy such as young female patients with the lower mediastinal disease.

## Figures and Tables

**Table 1:** Selection rules for Hodgkin lymphoma proton therapy based on the dosimetric comparison, currently in use at Institut Curie, Paris, France. The selection process relies on a competitive dosimetric comparison between VMAT and proton therapy, both simulated with deep-inspiration breath-hold (DIBH). Clinical characteristics of the patient are taken into account. One fulfilled criterion is sufficient. DIBH-VMAT: deep inspiration breath-hold volumetric modulated arc therapy. LVEF: left ventricle ejection fraction. DLCO: Diffusing capacity for carbon monoxide.

Criteria	Remark
Significant MHD reduction (vs. DIBH-VMAT)	>30% and >1 Gy
Significant mean breast dose reduction (vs. DIBH-VMAT)	>30% and >1 Gy, on one breast at least
Significant mean lung dose reduction (vs. DIBH-VMAT)	>50%, evaluated on all lungs simultaneously
History of mediastinal radiotherapy	
Genetic predisposition to breast cancer	
Baseline cardiac disease	LVEF reduction/coronaropathy
Baseline lung disease	DLCO decrease

## Abbreviations

CT	Compute tomography
DIBH	Deep inspiration breath-hold
EAR	Excess absolute risk
FB	Free-breathing
HL	Hodgkin lymphoma
IMPT	Intensity-modulated proton therapy
IMRT	Intensity-modulated radiation therapy
INRT	Involved-node radiation therapy
ISRT	Involved-site radiation therapy
MHD:	Mean heart dose
NTCP	Normal tissue complication probability
OAR	Organ-at-risk
RBE	Relative biological effectiveness
RR	Relative risk
r/r	Relapse/refractory
VMAT	Volumetric modulated arc therapy
